# Draft Genome Sequences of *Pseudomonas aeruginosa* Isolates from Wounded Military Personnel

**DOI:** 10.1128/genomeA.00829-16

**Published:** 2016-08-11

**Authors:** Brock A. Arivett, Dave C. Ream, Steven E. Fiester, Destaalem Kidane, Luis A. Actis

**Affiliations:** a Department of Microbiology, Miami University, Oxford, Ohio, USA; b Biology Department, Middle Tennessee State University, Murfreesboro, Tennessee, USA

## Abstract

*Pseudomonas aeruginosa*, a Gram-negative bacterium that causes severe hospital-acquired infections, is grouped as an ESKAPE (*Enterococcus faecium, Staphylococcus aureus, Klebsiella pneumoniae, Acinetobacter baumannii, Pseudomonas aeruginosa*, and *Enterobacter* species) pathogen because of its extensive drug resistance phenotypes and effects on human health worldwide. Five multidrug resistant *P. aeruginosa* strains isolated from wounded military personnel were sequenced and annotated in this work.

## GENOME ANNOUNCEMENT

*Pseudomonas aeruginosa* is a common environmental Gram-negative bacillus bacterium often associated with nosocomial infections including chronic lung infections in cystic fibrosis patients and bacteremia in burn victims. Human infections with *P. aeruginosa* can likely be traced back to 1862 when Luke observed rod-shaped particles in the blue-green pus of infections allowing this bacterium the opportunity to develop into a formidable human pathogen (1). Nosocomial pathogens, such as *P. aeruginosa*, have developed sophisticated resistance mechanisms since the introduction of antibiotics into the clinical setting (2). *P. aeruginosa* is currently the second most prevalent Gram-negative nosocomial pathogen preceded by *Escherichia coli* with as many as 2% of *P. aeruginosa* isolates specifically presenting with carbapenem-resistance (3). *P. aeruginosa* is referred to as an ESKAPE (*Enterococcus faecium, Staphylococcus aureus, Klebsiella pneumoniae, Acinetobacter baumannii, Pseudomonas aeruginosa*, and *Enterobacter* species) pathogen due to its ability to escape the lethal action of antibiotics (4). In order to develop a broader understanding of the mechanisms by which nosocomial *P. aeruginosa* strains escape death by antibiotics, the genome sequences of five *P. aeruginosa* strains isolated from wounded soldiers at the Walter Reed Army Medical Center (WRAMC) were determined using next-generation sequencing methods for future bioinformatic analyses.

Strains routinely stored at −80°C in 10% glycerol (5) were used to isolate total DNA from overnight LB cultures grown with agitation at 37°C using the DNeasy blood and tissue kit (Qiagen, Valencia, CA, USA). Absorption at 260 nm and 280 nm was measured for each sample to determine quantity and quality using the Nanodrop 2000 (Thermo Scientific, Wilmington, DE, USA). DNA concentrations for library preparation were determined by the SYBR green (Life Technologies, Grand Island, NY, USA) standard curve method in black 96-well plates (Corning, Tewksbury, MA, USA) using a FilterMax F5 spectrophotometer with Multi-Mode Analysis software version 3.4.0.25 (Molecular Devices, Sunnyvale, CA, USA). Whole DNA was sheared to approximately 500 bp in microTUBE-50 using M220 Focused-ultrasonicator (Covaris, Woburn, MA, USA). Fragmentation of resultant libraries was examined with a Bioanalyzer 2100 High Sensitivity DNA analysis kit (Agilent Technologies, Santa Clara, CA, USA) using version B.02.08.SI648 software. Individual libraries were normalized, pooled, and then sequenced using MiSeq v3 600-cycle kit (Illumina, San Diego, CA, USA) to perform 300-bp paired-end sequencing on a MiSeq instrument (Illumina) per manufacturer’s instructions. *De novo* assembly was performed using Genomics Workbench 8.0 with the bacterial genome finishing module (CLC bio, Boston, MA, USA) on a workstation with an AMD Opteron 2.10 GHz 16-core processor with 128 GB DDR3 ECC RAM. Genomes were annotated with Prokka version 1.10 on a quadcore i7 workstation with 32 GB DDR3 running Ubuntu 14.04 LTS (6). The *de novo* assembly statistics for the five *P. aeruginosa* sequenced isolates are shown in Table 1.

**TABLE 1:** Assembly metrics and accession numbers of *Pseudomonas aeruginosa* genomes

Strain ID	No. of contigs	*N*_50_ contigs (bp)	Total size (bp)	Coverage (×)	G+C content (%)	No. of ORFs^a^	No. of RNAs	Accession no.
105777	105	179,475	7,408,561	30	65.33	7,012	67	LODH00000000
105819	63	302,533	7,208,927	26	65.65	6,703	68	LOHH00000000
105880	86	215,191	6,914,271	17	65.98	6,490	60	LOHI00000000
105857	93	304,460	6,933,765	27	65.99	6,563	67	LOHJ00000000
105738	137	102,664	6,783,146	39	66.06	6,269	67	LOHK00000000

a Open reading frames.

### Accession number(s).

The whole-genome shotgun projects were deposited into GenBank under Bioproject ID PRJNA261239 with accession numbers listed in Table 1.
